# Suppressive effects of umbilical cord mesenchymal stem cell-derived exosomal miR-15a-5p on the progression of cholangiocarcinoma by inhibiting CHEK1 expression

**DOI:** 10.1038/s41420-022-00932-7

**Published:** 2022-04-15

**Authors:** Nuo Li, Baoming Wang

**Affiliations:** 1 Department of Gastroenterology, The Fourth Affiliated Hospital of China Medical University, Shenyang, 110000 P.R. China; 2 Department of Intervention, The Fourth Affiliated Hospital of China Medical University, Shenyang, 110000 P.R. China

**Keywords:** Cell biology, Diseases

## Abstract

Currently, surgical extraction is the main therapy for cholangiocarcinoma (CCA) patients, but it’s highly susceptible to postsurgical complications and recurrence rate. Thus, we identified the suppressing roles of exosomal miR-15a-5p from umbilical cord mesenchymal stem cells (UCMSCs) in the EMT and metastasis of CCA. The microarray dataset GSE265566 was employed to determine the expression of CHEK1 in CCA tissues. The relationship of miR-15a-5p with CHEK1 was analyzed using bioinformatics tools and dual-luciferase reporter assay. The particle size of HUCMSCs-exo was detected by scanning electron microscopy and nanoparticle tracking analysis. The cellular and tumorous phenotypes were assessed through flow cytometry, CCK-8 assay, Transwell assay and the in vivo tumor xenograft experiments. CHEK1 was predicated to be markedly elevated in CCA. miR-15a-5p targeted CHEK1 and downregulated the expression of CHEK1. HUCMSCs-exo activated cell apoptosis but repressed the proliferative, invasive, and migratory potentials of CCA cells. After miR-15a-5p was silenced, HUCMSCs-exo presented an opposite effect in regulating CCA. Overexpression of miR-15a-5p promoted apoptosis but suppressed malignancy and tumorigenicity of CCA cells as well as EMT through downregulating CHEK1. Our data suggested that miR-15a-5p in HUCMSCs-exo suppresses EMT and metastasis of CCA through targeting downregulation of CHEK1.

## Introduction

Cholangiocarcinoma (CCA) classified as intrahepatic cholangiocarcinoma (ICC) and extrahepatic cholangiocarcinoma (ECC) is a malignant tumor in the hepatobiliary system [1]. Clinical evidence shows that CCA is a rare form of highly heterogeneous and metastatic cancer [2]. The majority of CCA patients are often not diagnosed until advanced stages due to the lack of appropriate biomarkers [3]. Epidemiological data show that the incidence of CCA is raising globally, accounting for approximately 10–15% of all hepatobiliary carcinoma [4]. Currently, the therapy for CCA patients mainly relays on surgical extraction [5, 6]. However, the postsurgical complications and recurrence rate are high risk factors that severely affect the survival rate of CCA patients [6]. Clearly, novel therapeutic approaches are urgently needed for CCA patients [7]. More recently, mesenchymal stem cells (MSCs) have been discussed to play an important role in multiple malignant tumors [8, 9]. Given that MSCs are multipotent stem cells found to migrate towards and incorporate into the cancer cells [9], accumulating studies have shown that MSCs can mediate anti-tumor activity and inhibit the growth of tumor [10]. Importantly, as therapeutic carriers, MSCs provide precise location for drug delivery used in tumor-targeted therapy [11, 12].

MSCs have been confirmed to have a strong exosomal secretion capacity [13]. Exosomes are membrane vesicles that carry proteins, mRNAs and miRNAs and serve as potential tool for future anti-tumor therapies [9]. However, the therapeutic effect of MSCs-derived exosomes on CCA is still unclear and needs to be further investigated. Additionally, exosomal miRNAs derived from cancer stem cells have been implicated in controlling the development of malignant tumors [14, 15]. A recent study has highlighted the participation of miR-15a-5p in the metastasis and invasiveness of malignant tumors [16, 17]. Meanwhile, miR-15a-5p has been identified to serve as a tumor suppressor of human malignant tumor. However, there are few studies about the biological effects of miR-15a-5p on CCA. In our previous study, our gene-chip data showed that checkpoint kinase 1 (CHEK1) was highly expressed in CCA. CHEK1, as an oncogene activator, plays an essential role in tumorigenesis, including breast cancer, colon cancer and cervical cancer [18–20]. CHEK1 is also associated with an aggressive tumor phenotype and epithelial-mesenchymal transition (EMT) [19]. Thus, further understanding of the molecular mechanism of CHEK1 in CCA is of great importance. In this study, we aimed to uncover the mechanism by which exosomal miR-15a-5p prevents the progression of CCA.

## Results

### Bioinformatics analysis predicts the involvement of miR-15a-5p/CHEK1 in the progression of CCA

**Fig. 1:**
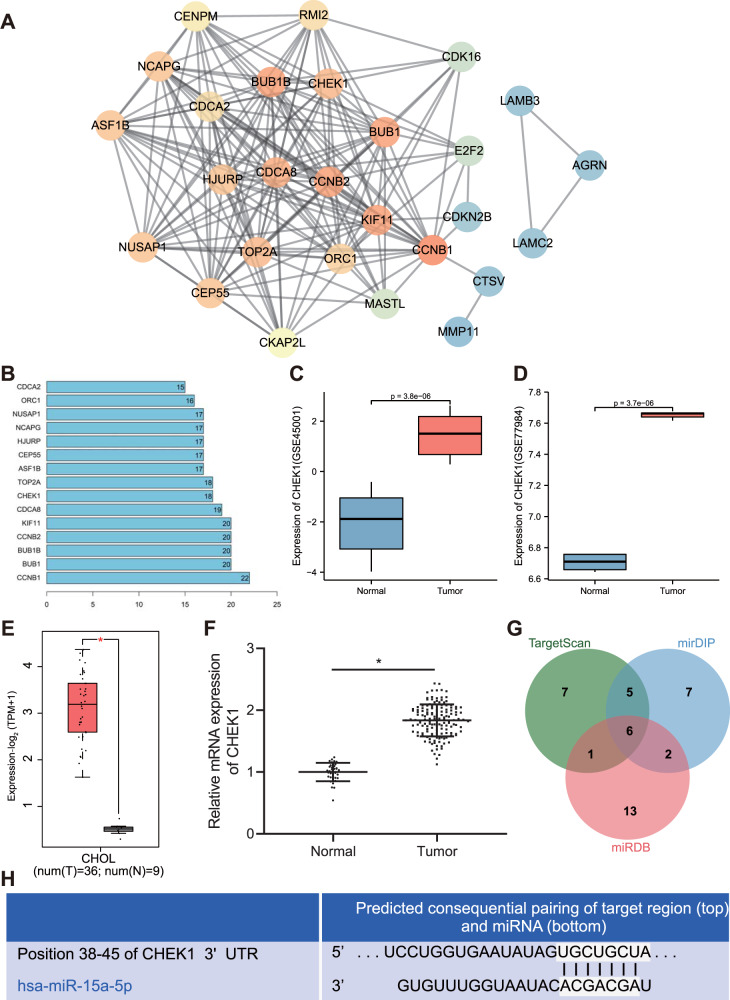
Bioinformatics analysis predicts the differentially expressed genes and their molecular interactions in CCA. **A** Interaction network of 50 differentially expressed genes in CCA-microarray GSE26566. Each cycle indicates a gene and the line between two cycles represents the interaction between two genes. More interaction genes of each gene show higher degree value, which display higher core degree with deeper color. **B** Degree value of the genes with a degree value higher than 15. The abscissa represents degree value and the ordinate represents gene names. **C** A box plot of CHEK1 expression in the GSE45001 microarray data (10 CCA samples and 10 normal samples). **D** A box plot of CHEK1 expression in the GSE77984 microarray data (3 CCA samples and 4 normal samples). **E** Differential expression of CHEK1 in CCA samples in TCGA and GTEx databases. The abscissa represents sample type and the ordinate represents gene expression. The red box indicates tumor sample and gray box indicates normal sample (*P < 0.01). **F** CHEK1 expression in CCA samples (*n* = 145) and normal samples (*n* = 35) determined by RT-qPCR. *means comparison against normal samples, *P* < 0.05. **G** Prediction of upstream miRNAs regulating CHEK1. The three circles in the figure represent the prediction results of the TargetScan, mirDIP, and miRDB databases, and the central part represents the intersection of the three databases. **H** The binding affinity of miR-15a-5p to CHEK1 as well as the binding site of miR-15a-5p in the 3’UTR of CHEK1 mRNA predicted by TargetscanHuman. The data are presented as mean ± standard deviation. Comparison between two groups was conducted using unpaired *t*-test.

Analysis of the CCA-related microarray data GSE26566 revealed 1273 differentially expressed genes in CCA samples, including 703 downregulated genes and 570 upregulated increased. We chose the top upregulated 50 genes for interaction analysis (Table S1), and constructed an interaction network as in Fig. 1A. Then, we counted the degree value of each gene in the interaction value, and 14 genes were found to have degree value higher than 15 (Fig. 1B). These 14 genes were further screened in GSE26566 microarray data. As displayed in Table S1, CHEK1 was the most significant upregulated gene. A published research has shown that CHEK1 is an important factor related to DNA replication and cell survival in cancer cells [21], and executes the function of the biological regulating enzyme in CCA [22]. The expression data of CHEK1 were further extracted from the CCA-related microarray data GSE45001 and GSE77984 and box plots were drawn. The results exhibited that CHEK1 was remarkably highly-expressed in the tumor samples (Fig. 1C, D). Next, the expression of CHEK1 in CCA samples and normal samples was evaluated in TCGA and GTEx databases, which consistently revealed the highly-expressed CHEK1 in CCA (Fig. 1E). Meanwhile, the level of CHEK1 was also upregulated in clinical samples of CCA (Fig. 1F). As shown in Table S2, the expression of CHEK1 was related to tumor size, lymph node metastasis and tumor stage (*P* < 0.05), but independent from gender and age of patients (*P* > 0.05).

To investigate the upstream mechanism of CHEK1, we used mirDIP, TargetScan and miRDB databases for prediction. Following Venn diagram analysis, 6 miRNAs were found at the intersection (Fig. 1G). miR-15a-5p was the miRNA with the highest score which interacted with CHEK1 mRNA in TargerscanHuman, with the predicted binding sites of the two shown in Fig. 1H. These results suggested that miR-15a-5p may participate in the progression of CCA by regulating CHEK1.

### miR-15a-5p targets CHEK1 and downregulates the expression of CHEK1

**Fig. 2:**
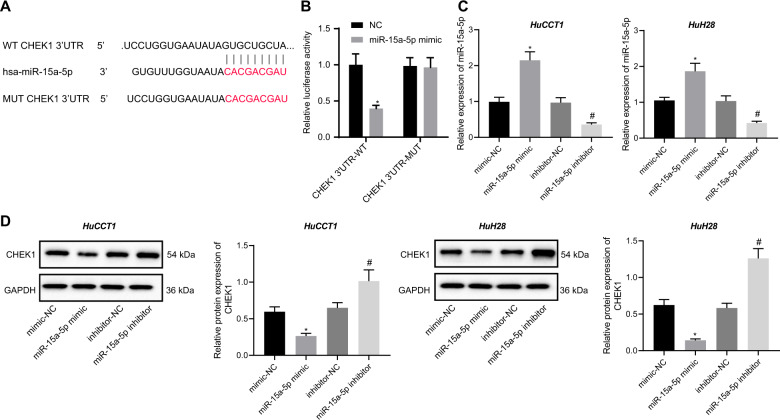
miR-15a-5p targets CHEK1 and downregulates CHEK1 expression. **A** Schematic diagram of miR-15a-5p binding sites in the 3′UTR of CHEK1 mRNA along with mutation site. **B** The relative luciferase activity of CHEK1-3′UTR-reporters responding to the miR-15a-5p mimic. The reporters were co-transfected with miR-15a-5p mimic into HEK-293T. Luciferase activity was tested after transfected for 48 h. **C** miR-15a-5p expression in HuCCT1 and HuH28 cells determined by RT-qPCR. **D** Western blot assay of CHEK1 protein in HuCCT1 and HuH28 cells. Quantitative results of protein bands are displayed at the right of the panel. In panels **C** and **D**, Mimic-NC, miR-15a-5p mimic, inhibitor-NC or miR-15a-5p inhibitor were individually transfected into HuCCT1 and HuH28 cells, RNA and protein levels of cells were analyzed after transfection for 48 h. The data are presented as mean ± standard deviation. Comparison between two groups was conducted using unpaired *t*-test, and the values at multiple groups were compared using one-way ANOVA followed by Tukey’s post-hoc tests. *means comparison against the NC or mimic-NC group, *P* < 0.05; ^#^means comparison against inhibitor-NC group, *P* < 0.05. The cell experiments were repeated three times independently.

Next, we aimed to validate the aforementioned involvement of the miR-15a-5p/CHEK1 in the progression of CCA. To explain the relationship of CHEK1 with miR-15a-5p, we constructed a wild-type reporter and a mutant reporter of 3’ UTR of CHEK1 mRNA using the luciferase-vector pRL-TK. Here, the wild type reporter (CHEK1 3′UTR-WT) contained a conservative recognized sequence of miR-15a-5p, and the mutant reporter (CHEK1 3′UTR-MUT) contained a mutated recognized sequence of miR-15a-5p (Fig. 2A). As shown in Fig. 2B, the relative luciferase activity of CHEK1 3′UTR-WT was reduced over but that of CHEK1 3′UTR-MUT showed no obvious changes upon transfection with miR-15a-5p mimic (Fig. 2B). Meanwhile, the results of RT-qPCR and Western blot assay revealed that compared to the mimic NC-treated HuCCT1 and HuH28 cells, miR-15a-5p mimic-treated HuCCT1 and HuH28 cells displayed increased levels of miR-15a-5p (Fig. 2C) but decreased mRNA and protein levels of CHEK1 (Fig. 2D). However, miR-15a-5p inhibitor-treated HuCCT1 and HuH28 cells displayed decreased levels of miR-15a-5p (Fig. 2C) but increased protein level of CHEK1 (Fig. 2D). These results demonstrate that miR-15a-5p targets CHEK1 and reduces the level of CHEK1.

### miR-15a-5p suppresses the malignant growth and EMT of CCA cells by targeting CHEK1

**Fig. 3:**
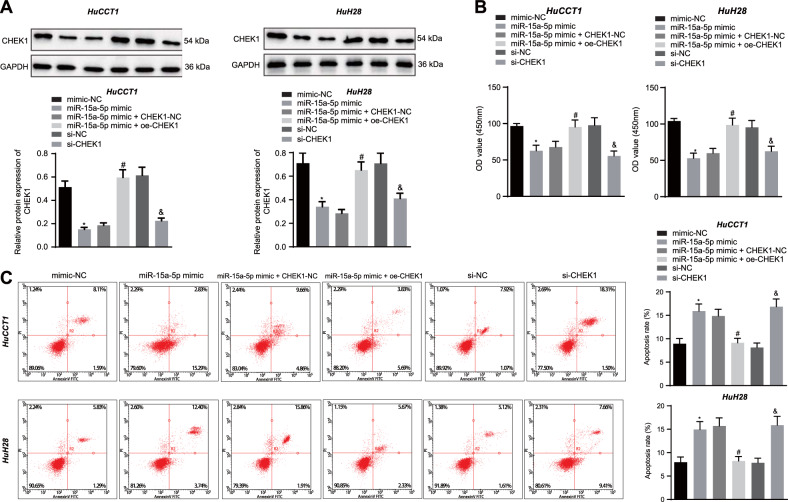
miR-15a-5p promotes apoptosis and represses the proliferation of CCA cells by targeting CHEK1. **A** Western blot assay of CHEK1 protein in HuCCT1 and HuH28 cells. Quantitative results of protein bands are displayed at the top of the panel. **B** Proliferative analysis of HuCCT1 and HuH28 cells. OD value was assessed at 450 nm. **C** Apoptosis evaluation of HuCCT1 and HuH28 cells using flow cytometry. In panels **A**–**C** miR-15a-5p mimic, miR-15a-5p mimic + oe-CHEK1, si-CHEK1, and their corresponding controls were individually transfected into HuCCT1 and HuH28 cells. The data are presented as mean ± standard deviation. The values at multiple groups were compared using one-way ANOVA followed by Tukey’s post-hoc tests; *means comparison against the mimic-NC group, *P* < 0.05; ^#^means comparison against miR-15a-5p mimic + CHEK1-NC group, *P* < 0.05; ^&^means comparison against the si-NC group, *P* < 0.05. Cell experiments were repeated three times independently.

**Fig. 4:**
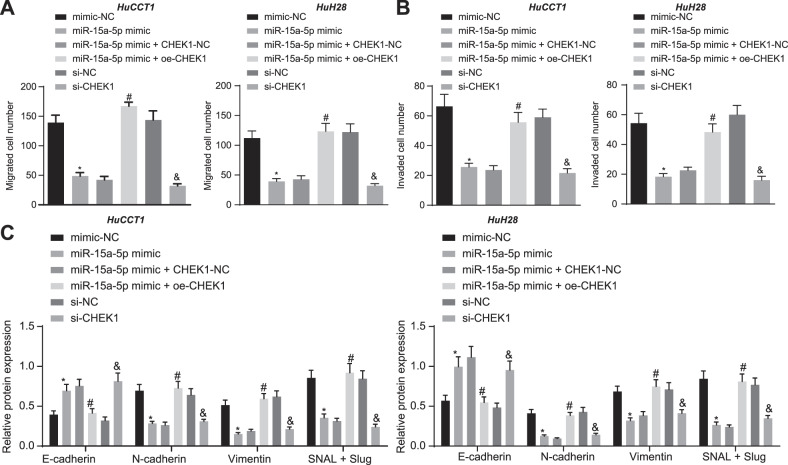
miR-15a-5p suppresses the migration, invasion and EMT of CCA cells by targeting CHEK1. **A** Transwell assay of migration of HuCCT1 and HuH28 cells. **B** Matrigel-based Transwell assay of invasion of HuCCT1 and HuH28 cells. **C** Western blot assay of EMT related protein markers. In panels A-C, miR-15a-5p mimic, miR-15a-5p mimic + oe-CHEK1, si-CHEK1, and their corresponding controls were individually transfected into HuCCT1 and HuH28 cells. Cell experiments were repeated three times independently. The data are presented as mean ± standard deviation. The values at multiple groups were compared using one-way ANOVA followed by Tukey’s post-hoc tests; *means comparison against the mimic-NC group, *P* < 0.05; ^#^means comparison against miR-15a-5p mimic + CHEK1-NC group, *P* < 0.05; ^&^means comparison against the si-NC group, *P* < 0.05.

In this part, we investigated whether miR-15a-5p regulates metastasis and EMT of CCA cells *via* targeting CHEK1 [23, 24]. Western blot assay results showed that the protein level of CHEK1 was reduced in HuCCT1 and HuH28 cells manipulated with miR-15a-5p mimic or si-CHEK1, while it was increased upon manipulation with miR-15a-5p mimic + oe-CHEK1 (Fig. 3A). In addition, flow cytometry, CCK-8 assay, and Transwell assay provided data suggesting attenuated proliferation, migration, and invasion yet an enhancement in apoptosis in HuCCT1 and HuH28 cells responding to miR-15a-5p gain-of-function or CHEK1 loss-of-function. These effects were reversed by CHEK1 re-expression (Fig. 3B, C, Fig. 4A, B, and Fig. S1A, S1B). Furthermore, as shown in Fig. 4C, E-cadherin expression was upregulated while expression of N-cadherin, Vimentin, and SNAIL/Slug was downregulated in miR-15a-5p mimic- or si-CHEK1-treated cells. The miR-15a-5p mimic-elicited alternation of the proteins was reversed by oe-CHEK1. According to the above results, we concluded that miR-15a-5p could diminish the expression of CHEK1 and repress the metastasis and EMT of CCA cells.

### miR-15a-5p overexpression suppresses the tumorigenesis by targeting CHEK1 in vivo

**Fig. 5:**
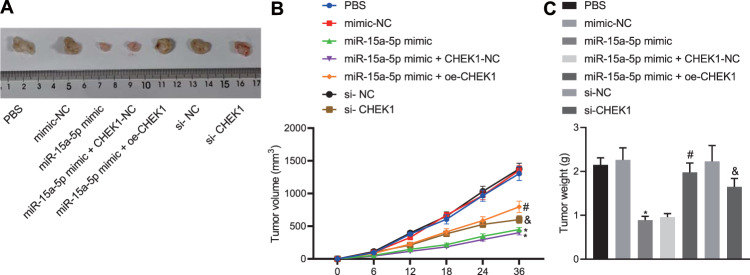
miR-15a-5p retards the tumorigenesis by inhibiting CHEK1 expression in vivo. **A** Images of the removed xenograft tumors. **B** Dynamic volume curve of xenograft tumors in vivo. **C** Weight of the removed xenograft tumors. In panels **A**–**C**, HuCCT1 cells treated by miR-15a-5p mimic, miR-15a-5p mimic + oe-CHEK1, si-CHEK1, their corresponding controls and PBS were individually subcutaneously injected into nude mice to construct nude mouse models of xenografts. The data are presented as mean ± standard deviation. Comparison to control group was conducted using paired *t*-test; the values at multiple groups were compared using one-way ANOVA followed by Tukey’s post-hoc tests; the values at different time points were compared using repeated measures ANOVA followed by Bonferroni pairwise comparison; *means comparison against the mimic NC group, *P* < 0.05. ^#^means comparison against miR-15a-5p mimic + CHEK1-NC group, *P* < 0.05; ^&^means comparison against the si-NC group, *P* < 0.05. *n* = 6 for mice in each group.

Now we turned to the experimental evidence on the in vivo tumor xenograft experiments. The HuCCT1 cells treated by miR-15a-5p mimic, miR-15a-5p mimic + oe-CHEK1, si-CHEK1, the corresponding controls and PBS control were subcutaneously injected to the BALB/c nude mice to establish the xenograft mice models. miR-15a-5p enhancement or CHEK1 silencing attenuated the tumorigenicity of CCA cells, accompanied by slow growth rate of the tumors. In the presence of miR-15a-5p, CHEK1 re-expression enhanced the tumorigenicity of CCA cells (Fig. 5A-C). Overall, these results indicate that miR-15a-5p can suppress tumorigenesis by inhibiting CHEK1 expression in vivo.

### miR-15a-5p is highly expressed in HUCMSCs and HUCMSCs-exo

**Fig. 6:**
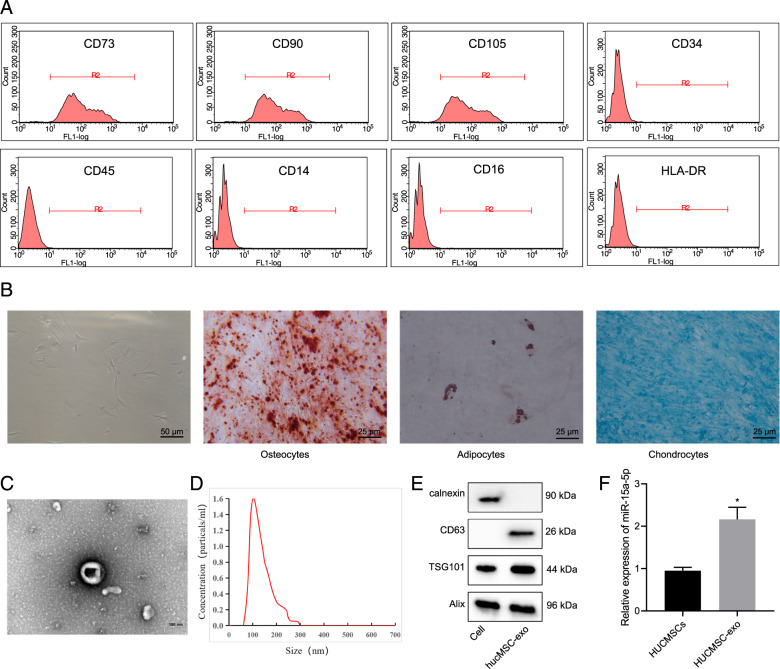
miR-15a-5p is highly expressed in HUCMSCs and HUCMSCs-exo. **A** Flow Cytometry detection of cell surface markers of HUCMSCs. **B** Observation of cell morphology and identification of differentiation-capacity under an inverted microscope. From left to right: Cell morphology was observed under an inverted microscope, bar = 50 μm; differentiation of osteoblasts was confirmed using Alizarin Red staining, bar = 25 μm; differentiation of adipocytes was assessed using Oil Red staining, bar = 25 μm; differentiation of chondroblasts was identified using Alcian Blue staining, bar = 25 μm. **C** TEM evaluation of exosome morphology. Bar = 100 μm. **D** NTA of particle size distribution of exosome. **E** Western blot assay of expression of the exosomal marker using ALIX, CD63 and TSG101 antibodies; calnexin antibody served as a NC. **F** Quantitative amplified results of level of miR-15a-5p in HUCMSCs and HUCMSCs-exo. Comparison to the control group was conducted using independent sample *t*-test; *means comparison against HUCMSCs, *P* < 0.05. The data are presented as mean ± standard deviation. Cell experiments were repeated three times independently.

Next, we isolated and purified HUCMSCs. Flow cytometric analysis suggested high positive rates (95%) of the surface markers of HUCMSCs (CD73, CD90, and CD105) while while positive rates of CD34, CD45, CD14, CD16, and HLA-DR under 2% in the purified samples (Fig. 6A). The results supported the isolated HUCMSCs were in high purity and could be applied in the following study. The purified HUCMSCs were fusiform- or spindle-shaped, clustering growth and forming spiral arrangement as observed under a light microscope, and they presented a good differentiation capacity of osteogenic, adipogenic, and chondrogenic in differentiation experiments (Fig. 6B).

Furthermore, we extracted exosomes from the supernatant of HUCMSCs at passage 2–4 using ultracentrifugation. TEM observation results showed that the exosomes were cystic and NTA analysis results indicated that distribution of the exosomes was within 100–200 nm (Fig. 6C, D). Furthermore, Western blot assay results revealed the presence of ALIX, CD63, and TSG101 expression in the extracted HUCMSC-exo, but no calnexin expression was observed (Fig. 6E). Our findings suggested the successful isolation of HUCMSC-exo that can be used in the following assay. As revealed by RT-qPCR, the level of miR-15a-5p was higher in HUCMSCs-exo than that in HUCMSCs. Cumulatively, miR-15a-5p was abundant in the HUCMSCs-exo (Fig. 6F).

### HUCMSCs-exo inhibits the malignant growth and EMT of CCA cells

**Fig. 7:**
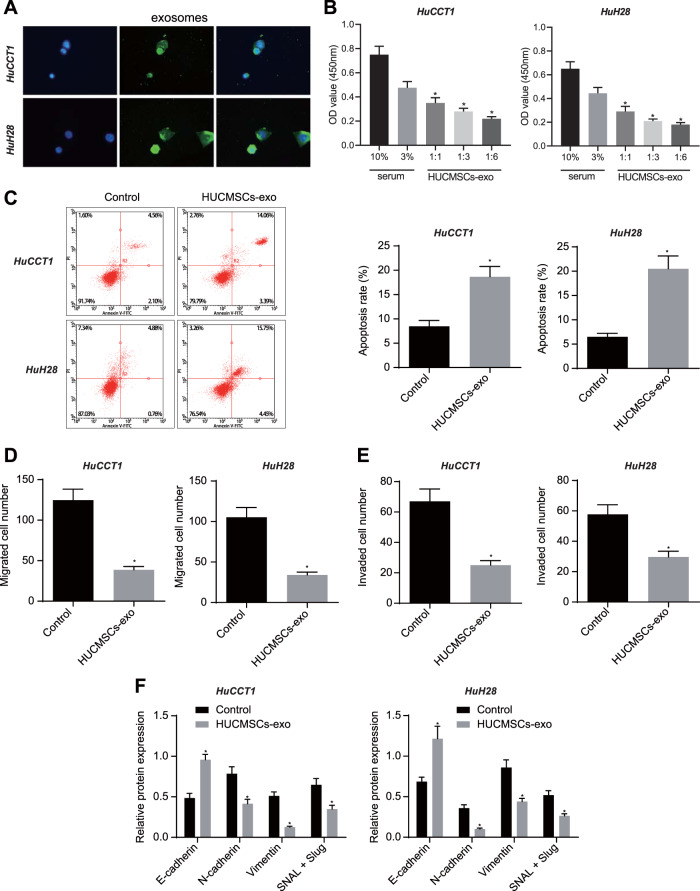
HUCMSCs-exo can be delivered to CCA cells to promote apoptosis, represses cell proliferation, migration, invasion, and EMT in CCA cells. **A** Fluorescence microscope observation of internalization of PKH67-labeled HUCMSCs-exo by HuCCT1 and HuH28 cells. **B** Proliferative analysis of HuCCT1 and HuH28 cells. OD value was assessed at 450 nm using the CCK-8 kit. **C** Apoptosis evaluation of HuCCT1 and HuH28 cells. The samples were tested using flow cytometry. Statistical results are exhibited at the right of the panel. **D** Transwell assay of migration of HuCCT1 and HuH28 cells. **E** Matrigel-based Transwell assay of invasion of HuCCT1 and HuH28 cells. **F** Western blot assay of EMT related protein markers. In panels **B**–**F**, HuCCT1 and HuH28 cells were treated by HUCMSCs-exo, with FBS as a control. Comparison with the control group was conducted using unpaired *t*-test; the values at multiple groups were compared using one-way ANOVA followed by Tukey’s post-hoc tests; * means comparison against the control group, *P* < 0.05. The data are presented as mean ± standard deviation. Cell experiments were repeated three times independently.

PKH67-labeled HUCMSCs-exo was incubated with CCA cells for 4 h. Fluorescence analysis showed the presence of PKH67 in the co-incubated HuCCT1 and HuH28 cells, suggesting that HUCMSCs-exo could be endocytosed (Fig. 7A). After HUCMSCs-exo treatment, we assessed the proliferation of CCA cells using CCK-8 kit. As shown in Fig. 7B, the proliferation rate was dependent on the dose of HUCMSCs-exo in both of HuCCT1 and HuH28 cells. Consistent with the above results, HUCMSCs-exo accelerated the cell apoptosis (Fig. 7C). In Transwell assay, both HuCCT1 and HuH28 cells co-cultured with HUCMSCs-exo for 24 h demonstrated decreased migration and invasion (Fig. 7D, E, Fig. S1C, S1D) [24]. Furthermore, the treated cells showed a high level of epidermal cell marker, E-cadherin, but low levels of mesenchymal cell markers, N-cadherin, Vimentin, and SNAIL/Slug (Fig. 7F). In short, HUCMSCs-exo can be delivered to CCA cells whereby limiting the proliferation, invasiveness, and EMT of CCA cells.

### miR-15a-5p in HUCMSCs-exo suppresses the malignancy and EMT of CCA cells

**Fig. 8:**
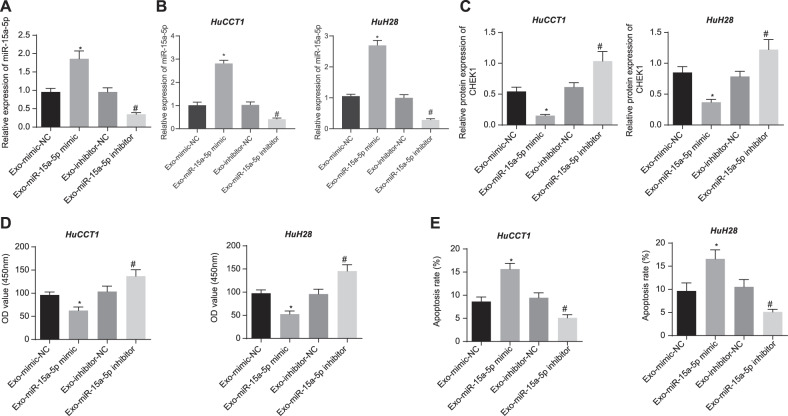
miR-15a-5p in HUCMSCs-exo promotes apoptosis and represses the proliferation of CCA cells *via* downregulation of CHEK1. **A** miR-15a-5p expression in HUCMSCs-exo determined by RT-qPCR. HUCMSCs-exo was sourced from HUCMSCs manipluated with mimic-NC, miR-15a-5p mimic, inhibitor-NC, or miR-15a-5p inhibitor. **B** miR-15a-5p expression in HuCCT1 and HuH28 cells determined by RT-qPCR. **C** Western blot assay of CHEK1 protein in HuCCT1 and HuH28 cells. **D** Proliferative analysis of HuCCT1 and HuH28 cells. OD value was assessed at 450 nm. **E** Apoptosis evaluation of HuCCT1 and HuH28 cells using flow cytometry. **B**–**E** Exo-miR-15a-5p mimic or Exo-miR-15a-5p inhibitor was individually transfected into CCA cells. The values at multiple groups were compared using one-way ANOVA followed by Tukey’s post-hoc tests; *means comparison against Exo-mimic-NC group, *P* < 0.05; ^#^means comparison against Exo-inhibitor-NC group, *P* < 0.05. The data are presented as mean ± standard deviation. Cell experiments were repeated three times independently.

Finally, we purified the HUCMSCs-exo from HUCMSCs which had been treated with mimic-NC, miR-15a-5p mimic, inhibitor-NC, and miR-15a-5p inhibitor, respectively, designated as Exo-mimic-NC, Exo-miR-15a-5p mimic, Exo-inhibitor-NC, and Exo-miR-15a-5p inhibitor groups. RNA levels of miR-15a-5p in the purified HUCMSCs-exo were shown in Fig. 8A. HuCCT1 and HuH28 cells treated by Exo-miR-15a-5p mimic exhibited increased level of miR-15a-5p (Fig. 8B) yet decreased CHEK1 level (Fig. 8C). However, Exo-miR-15a-5p inhibitor treatment led to opposite results. In addition, Exo-miR-15a-5p mimic repressed the proliferative, migratory and invasive functions of CCA cells while stimulating their apoptosis. Conversely, Exo-miR-15a-5p inhibitor induced contrary results (Figs. 8D, E, Fig. S2A, S1B, and Fig. S1E, S1F). Western blot assay results suggested that, after treatment with Exo-miR-15a-5p mimic, E-cadherin expression was promoted, and N-cadherin, Vimentin, SNAIL and Slug expression was diminished in CCA cells. Exo-miR-15a-5p inhibitor led to contrasting results (Fig. S2C). Overall, miR-15a-5p delivered by HUCMSCs-exo could suppress the proliferation, invasiveness, and EMT of CCA cells through downregulation of CHEK1.

## Discussion

CCA is a highly malignant tumor, lack of specific diagnostic biomarkers and clinical symptoms of most CCA patients are not evident until advanced disease [3]. Therefore, an exploitation of early diagnostic biomarkers and drug and/or molecular targets for CCA is imperative in clinic. Initially, our study showcased that CHEK1 mRNA and protein expression was significantly up-regulated in CCA tissues. CHEK1 is a DNA damage sensor that modulates cell-cycle progression, DNA damage response, and DNA replication [25–27]. Recent studies have revealed that CHEK1 is an oncogene in malignant tumor and plays an essential role in tumorigenesis, particularly in cancer prognosis and tumor phenotype [18, 19]. In our study, high expression of CHEK1 in CCA was dramatically correlated with tumor biological characteristics, such as tumor size, lymph node metastasis, cancer cell proliferation, invasion and migration, and EMT. Similarly, Roger, et al. reported that small-molecule CHEK1 inhibitors could effectively inhibit cancer cell proliferation in human lung and colorectal cancers [28]. Our data also revealed that silencing of CHEK1 in vitro appreciably impeded CCA cell proliferation, invasiveness and migration, and reversed EMT. In addition, silencing of CHEK1 in vivo could effectively decrease tumor size in nude mice. Clinical data validate that CHEK1 siRNA and chemical inhibitors are anticancer drug targets [29]. Furthermore, CHEK1 inhibition has the therapeutic potential in the cancer cell lines and mouse model [30, 31]. The above findings suggested that CHEK1 protein might be a biomarker to distinguish CCA and non-tumor patients.

We also found that overexpressed miR-15a-5p was capable of suppressing the growth of transplanted tumors. A previous study has reported that miR-15a-5p overexpression suppresses proliferative and dividing abilities of hepatocellular carcinoma cells [16]. miR-15a-5p also acts as a tumor suppressor in melanoma cells and restrains the viability and invasion of melanoma cells by muti-targeting genes [32]. miR-15a-5p overexpression decelerates endometrial cancer cell growth by directly targeting Wnt3a [17]. Moreover, miR-15a-5p blocks neuroblastoma progression by directly targeting v-myc myelocytomatosis viral related oncogene, neuroblastoma derived (avian) (MYCN) [33]. The above studies indicated that miR-15a-5p can be applied as a potential therapeutic target in human malignant tumor. The data of our study also proved that miR-15a-5p was enriched in exosomes secreted from HUCMSCs. Based on a previous study, HUCMSCs-exo harboring miR-148b-3p could delay breast cancer progression through down-regulating TRIM59 [34]. Also, HUCMSCs-derived exosomal miR-145-5p inhibits pancreatic ductal adenocarcinoma cell proliferation, invasiveness, and their resistance to apoptosis by down-regulating Smad3 expression [35]. These data consistently suggest that HUCMSCs-derived miRNAs can be used as novel prognostic biomarkers in many malignancies. In our study, exosomal miR-15a-5p derived from HUCMSCs obviously reduced CCA cell proliferation, invasion and migration, and decreased EMT markers protein expression.

Moreover, our findings indicated that exosomal miR-15a-5p originated from HUCMSCs exerted its functional effects on CCA by directly targeting CHEK1. Previous studies have shown that miR-15 family has an effect on the sensitivity to chemotherapy and resistance to radiation by controlling CHEK1 expression [36, 37]. In addition, other miRNAs, including miR-195, miR-497 and miR-424, can suppress the tumorigenesis by targeting CHEK1 [38–40]. Gemcitabine induces differential activation of CHEK1 in CCA cells and CHEK1 is responsible for the resistance of CCA cells to gemcitabine [41]. Small insertions and deletions (indels) of CHEK1 are present at the same genome positions among all types of CCA [42]. Encouragingly, our data validated that miR-15a-5p suppressed the development of CCA by directly targeting CHEK1. Specifically, HUCMSCs-exo carrying miR-15a-5p was identified to inhibit malignant progression, while repressing cell apoptosis in CCA by targeting CHEK1.

Collectively, our findings suggested important roles of the miR-15a-5p/CHEK1 axis in CCA progression (Fig. S3). Meanwhile, our finding of HUCMSCs-exo expressing miR-15a-5p yielded promising options that miR-15a-5p might be a potential therapeutic strategy as a CCA. However, the research is still at the preclinical stage. Additionally, the relevant intrinsic mechanisms of miR-15a-5p in CCA remains to be elaborated in future.

## Materials and methods

### Bioinformatics analysis

Data of CCA-related microarray data (GSE26566), with 169 samples (CCA sample: 104 cases; adjacent tissue: 59 cases; normal tissue: 6 cases, served as control), was downloaded from GEO database, and carried out a differential analysis with a selected limitation of logFC > 2 and FDR < 0.05 to adjust *P* value. CCA-related microarray data GSE45001 (10 CCA samples and 10 normal samples) and GSE77984 (3 CCA samples and 4 normal samples) were retrieved from the GEO database. Expression data of key genes were extracted separately for Weltch t’-test and a value of *p* < 0.05 indicates significant difference between normal and tumor samples. Then, STRIGN database was employed for interaction analysis of candidate upregulated genes, with gene interaction network constructed by cytoscape v3.7.1. The expression of CHEK1 in the CCA sample and normal sample in the dataset of TCGA and GTEx was evaluated in GEPIA2 database. Prediction in microRNAs which targets CHEK1-3′UTR was carried out by mirDIP, TargetscanHuman, and miRDB. The miRNAs with score higher than 0.5 in mirDIP, predicted as target miRNA in Conserved sites in TargetscanHuman, and with score higher than 90 in miRDB were selected, followed by intersection. After that, the binding site diagram of CHEK1 was obtained from Intersection. Finally, the network of miR-15a-5p in CCA was predicted.

### Clinical study

CCA clinical samples were collected from 145 patients (98 males and 47 females; aged 58.21 ± 8.11 years), who were diagnosed as CCA using histopathological examination at The Fourth Affiliated Hospital of China Medical University between January 2009 and December 2014. Among these patients, 45 patients presented with lymph node metastasis, and 100 patients without. According to tumor-node-metastasis (TNM) staging of the American Joint Committee on Cancer (AJCC)/International Union Against Cancer (UICC), 109 cases belonged to the TI stage, 30 cases belonged to the TII stage, and 6 cases belonged to the TIV stage. Additionally, 35 cases from normal bile duct after liver transplantation were enlisted as a control, and all samples were saved in liquid nitrogen before extracting protein [23]. This study was approved by the Ethics Committee of The Fourth Affiliated Hospital of China Medical University, and adhered to the tenets of the *Declaration of Helsinki*. All patients had signed informed consent prior to enrollment.

### Isolation, culture and identification of HUCMSCs

Isolation and culture of HUCMSCs: after approval of the Ethics Committee of The Fourth Affiliated Hospital of China Medical University, the human umbilical cords used in this study were isolated from 3 pregnant women (aged 25−27 years, had signed the informed consent) of term cesarean section at The Fourth Affiliated Hospital of China Medical University. The isolated human umbilical cords were dealt with in the best 6 h [43]. Umbilical cords were rinsed until arteries removed using phosphate-buffered saline (PBS) contained 5% antibiotics (penicillin and streptomycin). The umbilical cords were spliced into small pieces (1–3 mm^3^) and resuspended in low-glucose Dulbecco’s modified Eagle’s medium (DMEM) (Gibco, Carlsbad, CA, USA) supplemented with 10% fetal bovine serum (FBS) and 1% penicillin and streptomycin. The cells were cultured at 37 °C in a 5% CO_2_-contained atmosphere and replaced with fresh medium every 3 days. The adherent cells with 80% density were digested using 0.25% trypsin (Invitrogen, Carlsbad, CA, USA) and passed with the expanded culture [44].

Identification of HUCMSCs: HUCMSCs at passage 2 were rinsed twice with PBS, resuspended and plated in a 6-well plate upon the 10^6^ cells/mL density, followed by staining as per the fluorescent antibody instructions. The assay of flow cytometry was operated utilizing primary antibodies of FITC-CD34, CD71, HLA-DR, PE-CD29, CD38, CD44, CD105, and HLA-I, with PE-IgG1 and FITC-IgG1 as control antibodies. The above antibodies were purchased from BD Biosciences (San Jose, CA, USA). The flow cytometry was analyzed *via* the FACSAria II Special Order System (BD Biosciences), and the data of flow cytometry were processed by software FlowJo [43].

The HUCMSCs at passage 3 were carried out evaluations of differentiation capacity through the medium of osteogenic, adipogenic, and chondrogenic following the user manual of the medium (Cyagen Biosciences, Guangzhou, China), and assessed using dyes of Alizarin Red, Oil Red and Alcian Blue [45].

### Cell culture

Human CCA cell lines, HuCCT1, and HuH28 (BNCCS, Beijing, China) were cultured in RPMI 1640 medium with 10% FBS, 100 U/mL penicillin and 100 ug/mL streptomycin at 37 °C in a 5% CO_2_ incubator.

### Isolation, identification, and labeling of HUCMSCs-exo

The medium of the HUCMSCs at passage 3 was collected and ultracentrifuged to isolate exosomes. The collected cell medium was centrifuged at 1000 g for 20 min to remove the cell debris followed by centrifugation at 2000 g for 20 min and 10,000 g for 30 min. The supernatant was collected and concentrated in MWCO tubes (MW: 100 KDa, Millipore, USA) at 1000 g for 30 min. The concentrated supernatant was ultracentrifuged at 100,000 g for 60 min in 30% sucrose/D_2_O buffer, using Optimal-90k (Beckman Coulter, Brea, CA, USA). The exosome-enriched fraction was collected from the lower parts of the ultracentrifuged sample and diluted with PBS, and then centrifuged three times at 1,000 g for 30 min using 100 KDa MWCO tube. Finally, the purified exosomes (HUCMSCs-exo) were filtered by a 0.22 μm pore filter (Millipore, Billerica, MA, USA) and stored at −80 °C [44].

HUCMSCs-exo were observed using transmission electron microscopy (TEM) (FEI Tecnai 12, Philips, Netherlands). Concentration and size of the HUCMSCs-exo were detected in a 1:10 dilution by nanoparticle tracking analysis (NTA) in the NanoSight NS500 system (Malvern, Westborough, MA, USA). After lysed in RIPA buffer, the concentration of exosomal protein was determined using a BCA protein assay kit (Pierce, Rockford, IL, USA). Antibodies of ALIX (ab88743, Abcam, Cambridge, UK), CD63 (ab216130, Abcam), TSG101 (ab30871, Abcam) and calnexin (ab10286, Abcam) were applied in Western blot assay [44].

Labeling carried out via staining HUCMSCs-exo (1 mL) with 5 μL PKH67 (Molecular Probes, Invitrogen, Carlsbad, CA, USA) for at 37 °C for 30 min. After stained, removal of excess dye was achieved by washing with PBS and centrifugation at 100,000 g for 1 h [46]. In the in vitro tracking experiment, we incubated PKH67-labeled HUCMSCs-exo with CCA cells for 4 h and observed under an Olympus BX41 microscope equipped with CCD (MagnaFire, Olympus, Tokyo, Japan) [44].

### Transfection

Mimic-NC, miR-15a-5p mimic, inhibitor-NC, miR-15a-5p inhibitor [47], CHEK1-NC, oe-CHEK1, si-NC and si-CHEK1 were purchased from Guangzhou RiboBio Co., Ltd. (Guangzhou, China).

Cells were plated into a 6-well plate with a density of 3 × 10^5^ cells/well and cultured for about 24 h, till cell confluence reached 80%. The prepared cells were transfected using Lipofectamine 2000 reagent (Invitrogen), following the procedure recommended by the manufacturer. Additionally, the transfected samples were added into the medium with a final concentration of 60 nM.

After transfected for 48 h, the cells were harvested and the transfection efficiency was determined using RT-qPCR or Western blot assay.

### RT-qPCR

Total RNA was extracted from cultured cells or tissues using the TRIzol reagent (Invitrogen). For mRNA detection, reverse transcription kit (RR047A, Takara, Japan) was adopted to obtain cDNA. MicroRNAs in medium (350 μL) and EVs (100 μg) were extracted using mirVana PARIS Kit (Ambion, Naugatuck, CT, USA), with cel-miR-39 (1 pmol per sample; TianGen, Beijing, China) as an exogenous reference. For miRNA detection, microRNA Reverse Transcription Kit (EZB-miRT2-S, EZBioscience, USA) was applied for generating cDNA. microRNA-cDNAs were quantitatively amplified utilizing miRcute Plus miRNA qPCR Detection Kit (TianGen) with U6-cDNA as an internal reference. GAPDH was regarded as the internal reference of mRNA. The expression of each gene or isoform was quantified using the comparative threshold method with the formula of 2^−ΔΔCt^. The primer sequences of quantitative amplification were listed below Table S3 [48].

### Western blot assay

The transfected cells were collected after culturing for 48 h. Washed in PBS once, the collected cells were lysed in RIPA buffer (P0013B, Beyotime, Shanghai, China) with PMSF, incubated on ice for 30 min, and centrifuged at 4 °C at 12,000 rpm for 10 min. Total protein was calibrated to a concentration of 4 μg/μL using a BCA protein assay kit (Pierce, Rockford, IL, USA) and 30 μg was loaded for Western blot assay. Rabbit anti-human antibodies of CHEK1 (1:1,000, ab32531), E-cadherin (1:1,0000, ab40772), N-cadherin (1:1,000, ab18203), Vimentin (1:1,000, ab92547), SNAIL + Slug (1:2,000, ab180714), GAPDH (1:2,500, ab9485) and second goat anti-rabbit antibody labeled with HRP (1:2,000, ab205718) were purchased from Abcam, Cambridge, UK. Incubation with all primary antibodies was carried out at 4 °C overnight. The blots were visualized with SmartView Pro 2000 (UVCI-2100, Major Science, Saratoga, CA, USA) and protein bands were quantitated by ImageJ 1.48 u (National Institutes of Health, Bethesda, MD, USA) [49]. The experiments were repeated at least three times with similar results.

### Dual-luciferase reporter analysis

The synthesized fragment of CHEK1-3′UTR was constructed into pRL-TK (Promega, Madison, WI, USA). The seed sequence of miR-15a-5p in CHEK1-3′UTR was replaced to construct a mutant of the luciferase reporter. CHEK1 3′UTR-WT and CHEK1 3’UTR-MUT (100 ng) were individually co-transfected with miR-15a-5p mimic or mimic-NC into HEK-293T cells (4 × 10^5^ cells; CRL-1415, Xinyu Biotech., Shanghai, China). The transfected cells after culturing for 48 h were evaluated using the Luciferase assay kit (RG005, Beyotime, Shanghai, China) with Glomax20/20 luminometer (Promega) [50, 51]. The experiments were repeated at least three times with similar results.

### Proliferation assay

The cells transfected for 48 h were placed in a 96-wells plate with triple repeats for each sample, based on the density of 1 × 10^4^ cells/well. After 72 h of culture, proliferation assay of the treated cells was performed with CCK-8 assay (10 μL/well, Solarbio Biotech., Beijing, China) for 4 h [23]. The absorbance at 450 nm was determined using automatic Enzyme Labeling MK3 (Thermo Fisher, Waltham, MA, USA).

### Flow cytometry assay for cell apoptosis

The cells transfected for 48 h were replaced by a serum-free medium and incubated to induce apoptosis. The treated cells were digested with 0.25% trypsin and stained with propidium iodide (PI) and Annexin-V (Annexin V-FITC Apoptosis Detection Kit, Abcam) [23]. Flow cytometry experiments were carried out with FACSAria II Special Order System (BD Biosciences) and analyzed by FlowJo.

### Evaluation of migration and invasion using Transwell assay

HuCCT1 and HuH28 cells treated in serum-free medium for 24 h were digested and diluted to a concentration of 2 × 10^5^ cells/mL. The diluted cell suspension (200 μL) was plated in the upper chamber of the Transwell unit, 700 μL of cold DMEM containing 10% FBS was appended to the lower chamber of the Transwell unit, and the Transwell plate was incubated at 37 °C in a 5% CO_2_-contained atmosphere. After 24 h incubation, the Transwell units were removed, and the samples were fixed in 4% paraformaldehyde for 30 min, followed by 0.1% crystal violet staining for 20 min. The stained cells were counted in five random fields per well using the inverted microscope.

Matrigel gel was incubated at 4 °C overnight and diluted to 1 mg/mL with a serum-free medium. Then, 40 μL of the diluted Matrigel was plated to the upper chamber of the Transwell unit and incubated at 37 °C for 5 h for solidification. Matrigel invasion chambers were hydrated for 30 min before starting the invasion assay. HuCCT1 and HuH28 cells cultured in serum-free medium for 24 h were digested and diluted to a concentration of 2 × 10^5^ cells/mL. Diluted cell suspension (200 μL) was plated to the prepared upper chamber of the Transwell unit, 700 μL of cold DMEM containing 10% FBS was appended to the lower chamber, and the plate was incubated at 37 °C in a 5% CO_2_-contained atmosphere. After 24 h, the Transwell units were removed for 4% paraformaldehyde fixation for 30 min, and 0.1% crystal violet staining for 20 min. The stained cells were counted in five random fields per well using the inverted microscope.

### Xenograft tumor model in nude mice

HuCCT1 cell suspension (1 × 10^6^ cells, 100 μL) following manipulation with mimic NC, miR-15a-5p mimic, miR-15a-5p mimic + CHEK1-NC, miR-15a-5p mimic + oe-CHEK1, si-NC or si-CHEK1 was injected subcutaneously into the dorsal region of 42 BALB/c mice (male, aged 5 weeks, weighing 15−17 g; Beijing HFK Bio-Technology Co., LTD., Beijing, China). PBS-treated mice were used as the control. We recorded the tumor volume of nude mice per 6 days and finally removed the xenograft tumor for size assessment [52]. After 36 days, the mice were euthanized by carbon dioxide asphyxiation.

### Statistical analysis

Data were calculated as the mean ± standard deviation from at least three independent experiments. The statistical comparison was performed using unpaired *t*-test when only two groups were compared or by Tukey’s test-corrected one-way analysis of variance (ANOVA) with when more than two groups were compared. Comparison of data at different time points was performed utilizing Bonferroni-corrected repeated measures ANOVA. All statistical analyses were detected using SPSS 21.0 software (IBM Corp. Armonk, NY, USA) with *P* < 0.05 as a level of statistical significance.

## Supplementary information

Figure S1

Figure S2

Figure S3

Table S1

Table S2

Table S3

Original Data File

## Data Availability

The datasets generated and analyzed during the current study are available from the corresponding author on reasonable request.
