# Supplementary material for: Suppressive effects of umbilical cord mesenchymal stem cell-derived exosomal miR-15a-5p on the progression of cholangiocarcinoma by inhibiting CHEK1 expression
Source: Cell Death Discov. 2022 Apr 15;8:205. doi: 10.1038/s41420-022-00932-7 (PMC9012823; doi:10.1038/s41420-022-00932-7)
Supplement: Supplementary file 1 — Figure S1 [file 41420_2022_932_MOESM1_ESM.docx]

**
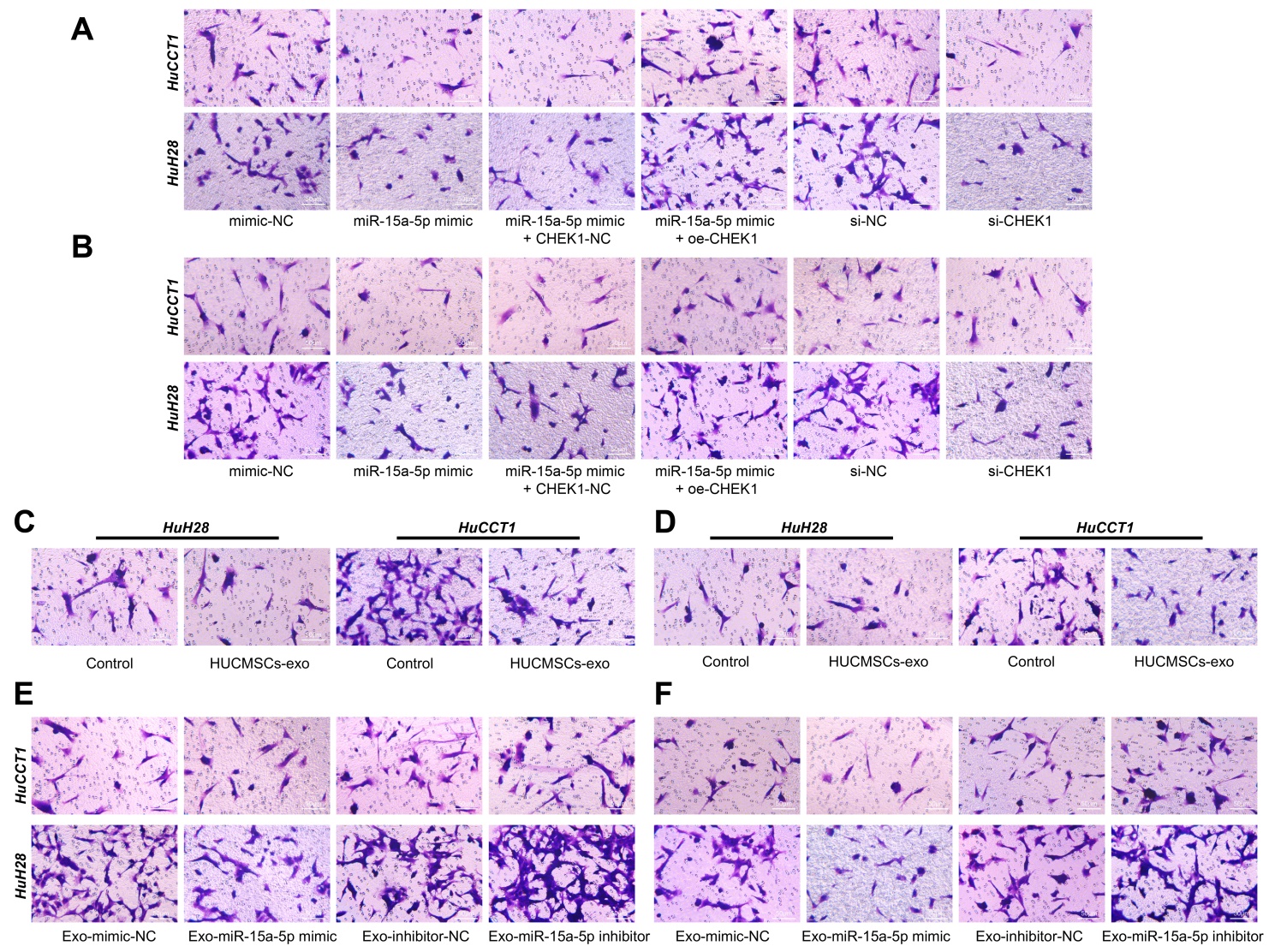
**

**Figure S1.** Representative images of Transwell migration and invasion assays. A-B, Representative images of migrated (A) and invaded (B) HuCCT1 and HuH28 cells treated with miR-15a-5p mimic, miR-15a-5p mimic + oe-CHEK1, si-CHEK1, and their corresponding controls. C-D, Representative images of migrated (C) and invaded (D) HuCCT1 and HuH28 cells were treated by HUCMSCs-exo, with FBS as control. E-F, Representative images of migrated (E) and invaded (F) HuCCT1 and HuH28 cells were treated by Exo-miR-15a-5p mimic or Exo-miR-15a-5p inhibitor.
