# Supplementary material for: Suppressive effects of umbilical cord mesenchymal stem cell-derived exosomal miR-15a-5p on the progression of cholangiocarcinoma by inhibiting CHEK1 expression
Source: Cell Death Discov. 2022 Apr 15;8:205. doi: 10.1038/s41420-022-00932-7 (PMC9012823; doi:10.1038/s41420-022-00932-7)
Supplement: Supplementary file 2 — Figure S2 [file 41420_2022_932_MOESM2_ESM.docx]

**
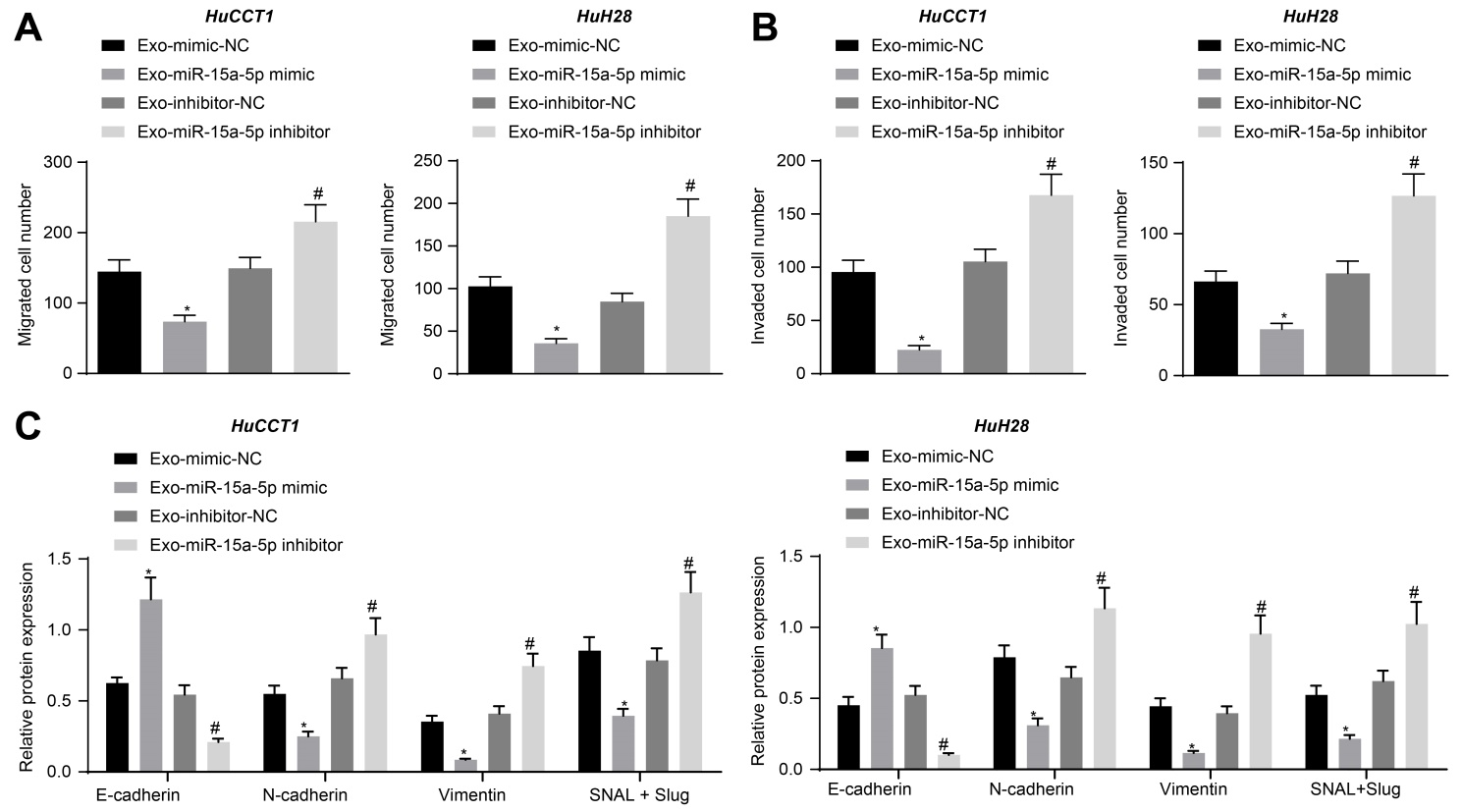
**

**Figure S2.** miR-15a-5p in HUCMSCs-exo represses the in vitro malignant progression and EMT through downregulation of CHEK1. A. Transwell assay of migration of HuCCT1 and HuH28 cells. B. Matrigel-based Transwell assay of invasion of HuCCT1 and HuH28 cells. C. Western blot assay of EMT-related protein markers. In panels A-C, HuCCT1 and HuH28 cells were treated by Exo-miR-15a-5p mimic or Exo-miR-15a-5p inhibitor. The values at multiple groups were compared using one-way ANOVA followed by Tukey’s post-hoc tests; * means comparison against Exo-mimic-NC group, P < 0.05; # means comparison against Exo-inhibitor-NC group, P < 0.05. The data are presented as mean ± standard deviation. Cell experiments were repeated three times independently.
