# Supplementary material for: Suppressive effects of umbilical cord mesenchymal stem cell-derived exosomal miR-15a-5p on the progression of cholangiocarcinoma by inhibiting CHEK1 expression
Source: Cell Death Discov. 2022 Apr 15;8:205. doi: 10.1038/s41420-022-00932-7 (PMC9012823; doi:10.1038/s41420-022-00932-7)
Supplement: Supplementary file 3 — Figure S3 [file 41420_2022_932_MOESM3_ESM.docx]

**
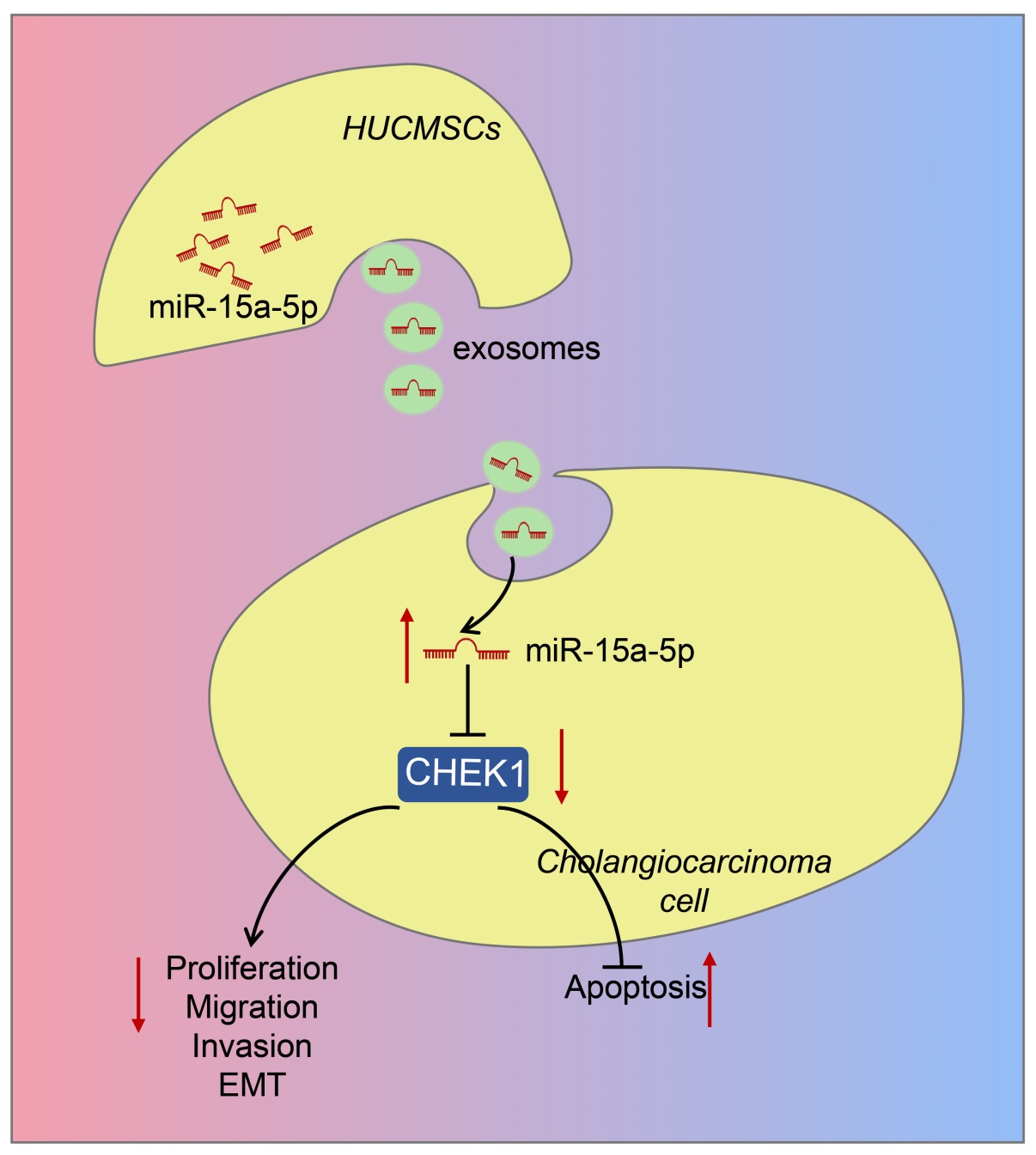
**

**Figure S3.** Schematic diagram of the mechanism by which miR-15a-5p in HUCMSCs-exo affects the progression of CCA. HUCMSCs-exo deliver miR-15a-5p to CCA cells wherein miR-15a-5p targets and down-regulates the expression of CHEK1, thereby impairing the proliferation, invasion, migration, and EMT of CCA cells, and promoting their apoptosis, ultimately inhibiting the progression of CCA.
