# Supplementary material for: Suppressive effects of umbilical cord mesenchymal stem cell-derived exosomal miR-15a-5p on the progression of cholangiocarcinoma by inhibiting CHEK1 expression
Source: Cell Death Discov. 2022 Apr 15;8:205. doi: 10.1038/s41420-022-00932-7 (PMC9012823; doi:10.1038/s41420-022-00932-7)
Supplement: Supplementary file 4 — Table S1 [file 41420_2022_932_MOESM4_ESM.docx]

**Table S1** Interaction analysis of the top upregulated 50 genes in CCA samples

| Symbol | logFC | AveExpr | t | *p* value | adj.P.Val | B |
| --- | --- | --- | --- | --- | --- | --- |
| TIMM10 | 2.42402841 | 8.79239248 | 8.107880991 | 7.54E-13 | 6.01E-10 | 18.80610365 |
| FGD6 | 4.290882807 | 6.294474843 | 7.834036899 | 3.08E-12 | 2.16E-09 | 17.45559626 |
| LOC400506 | 2.405683762 | 5.320697757 | 7.610038813 | 9.67E-12 | 5.65E-09 | 16.3600077 |
| CHEK1 | 4.629355134 | 5.503212095 | 7.191389645 | 9.16E-11 | 4.46E-08 | 14.21066669 |
| BUB1B | 5.83472334 | 5.735900336 | 7.211790222 | 1.22E-10 | 5.79E-08 | 13.41860265 |
| CST2 | 5.193858426 | 5.834716479 | 6.797854042 | 5.59E-10 | 2.18E-07 | 12.47411324 |
| MASTL | 2.574409766 | 5.458361453 | 6.699882308 | 9.01E-10 | 3.44E-07 | 12.01629351 |
| OSBPL3 | 2.629252733 | 4.82717784 | 6.690686678 | 9.69E-10 | 3.61E-07 | 11.94880241 |
| DTX2 | 2.689851515 | 5.423049966 | 6.446258939 | 3.15E-09 | 9.14E-07 | 10.82218599 |
| CKAP2L | 4.084980209 | 5.673950393 | 6.450522256 | 3.16E-09 | 9.14E-07 | 10.81644609 |
| ORC1L | 2.978396427 | 5.304322985 | 6.444162239 | 3.18E-09 | 9.14E-07 | 10.81259902 |
| CENPM | 3.876666838 | 6.763963771 | 6.335356217 | 5.22E-09 | 1.37E-06 | 10.33750449 |
| MEST | 2.646723027 | 3.932692164 | 6.327712511 | 5.41E-09 | 1.40E-06 | 10.30274382 |
| CAPG | 2.839265183 | 7.417559593 | 6.318835256 | 5.64E-09 | 1.43E-06 | 10.26239733 |
| LAMC2 | 4.574205608 | 8.425700586 | 6.273468358 | 7.00E-09 | 1.73E-06 | 10.05661168 |
| PEX6 | 2.633731264 | 6.724879155 | 6.225984743 | 8.77E-09 | 2.11E-06 | 9.841956854 |
| KIF11 | 3.635478526 | 5.867006202 | 6.194229864 | 1.02E-08 | 2.35E-06 | 9.698829769 |
| B3GNTL1 | 3.910287694 | 4.309805008 | 6.215259186 | 1.03E-08 | 2.35E-06 | 9.697450651 |
| DKFZp762E1312 | 2.837720598 | 5.890302016 | 6.096991218 | 1.61E-08 | 3.48E-06 | 9.262711405 |
| C11orf49 | 2.58598997 | 3.019817025 | 6.107101325 | 1.82E-08 | 3.85E-06 | 9.160676582 |
| CCNB1 | 3.479469622 | 5.818508208 | 5.975587962 | 3.00E-08 | 5.78E-06 | 8.671797358 |
| GJB3 | 7.112328547 | 5.535858914 | 5.996457632 | 2.96E-08 | 5.76E-06 | 8.641144695 |
| NCAPG | 4.014559348 | 6.56998291 | 5.929260332 | 3.57E-08 | 6.67E-06 | 8.512389747 |
| MMP11 | 6.184118119 | 9.16366682 | 5.926784962 | 3.68E-08 | 6.80E-06 | 8.474581506 |
| SIGLEC10 | 3.289199224 | 5.023953337 | 5.90837872 | 4.01E-08 | 7.32E-06 | 8.399574072 |
| BUB1 | 3.163799384 | 6.409940443 | 5.897957996 | 4.13E-08 | 7.44E-06 | 8.375697638 |
| CDKN2B | 4.29776388 | 4.423974397 | 5.883832769 | 4.84E-08 | 8.18E-06 | 8.226216154 |
| C14orf151 | 2.493580495 | 5.489851125 | 5.752988028 | 7.88E-08 | 1.22E-05 | 7.747477425 |
| SLC22A18AS | 3.577774126 | 6.813993778 | 5.733992549 | 8.59E-08 | 1.31E-05 | 7.665129874 |
| C16orf75 | 2.787000811 | 7.431979138 | 5.724312979 | 8.98E-08 | 1.36E-05 | 7.623223268 |
| NUSAP1 | 2.302652218 | 8.432061716 | 5.701240269 | 9.97E-08 | 1.48E-05 | 7.523484303 |
| KIAA1189 | 2.920228219 | 3.155622295 | 5.660933299 | 1.28E-07 | 1.81E-05 | 7.292968054 |
| LAMB3 | 4.260866445 | 7.162340347 | 5.643772824 | 1.31E-07 | 1.84E-05 | 7.262279354 |
| CEP55 | 6.37439177 | 6.384611803 | 5.643925015 | 1.45E-07 | 2.02E-05 | 7.168157799 |
| ADAM8 | 4.047108886 | 6.405017828 | 5.574275066 | 1.77E-07 | 2.37E-05 | 6.978528613 |
| MAL2 | 2.60767708 | 10.19571144 | 5.571412996 | 1.79E-07 | 2.38E-05 | 6.966321268 |
| JARID1C | 3.211462613 | 4.343486085 | 5.530150399 | 2.29E-07 | 2.91E-05 | 6.738469055 |
| CDCA8 | 4.368433488 | 5.641201597 | 5.46867279 | 2.91E-07 | 3.62E-05 | 6.537270784 |
| CTSL2 | 4.035469582 | 6.859421064 | 5.472998747 | 2.82E-07 | 3.53E-05 | 6.536392459 |
| TOP2A | 3.841475168 | 8.032198331 | 5.43457709 | 3.29E-07 | 4.04E-05 | 6.386766034 |
| AGRIN | 2.1838051 | 9.602933612 | 5.412009663 | 3.64E-07 | 4.31E-05 | 6.291964468 |
| CDCA2 | 3.040199777 | 3.878081907 | 5.393874316 | 4.17E-07 | 4.90E-05 | 6.168200132 |
| PAQR5 | 3.985376296 | 5.071156569 | 5.3685173 | 4.53E-07 | 5.29E-05 | 6.086798402 |
| E2F2 | 2.842355056 | 6.133773465 | 5.354708836 | 4.75E-07 | 5.47E-05 | 6.040916697 |
| ASF1B | 3.874975753 | 5.787942233 | 5.357967816 | 4.94E-07 | 5.52E-05 | 6.030782363 |
| PAFAH1B3 | 2.077343332 | 7.37178302 | 5.345376377 | 4.88E-07 | 5.49E-05 | 6.013369916 |
| CCNB2 | 3.477537075 | 7.288720781 | 5.325650046 | 5.32E-07 | 5.87E-05 | 5.931276214 |
| PCTK1 | 2.120122484 | 2.962639772 | 5.306180217 | 6.28E-07 | 6.79E-05 | 5.781858889 |
| ENTPD6 | 3.622418785 | 4.608758203 | 5.291212316 | 6.61E-07 | 7.10E-05 | 5.77346929 |
| C2orf27 | 2.538294308 | 4.52561985 | 5.276076907 | 6.69E-07 | 7.11E-05 | 5.714997509 |
