# Supplementary material for: Suppressive effects of umbilical cord mesenchymal stem cell-derived exosomal miR-15a-5p on the progression of cholangiocarcinoma by inhibiting CHEK1 expression
Source: Cell Death Discov. 2022 Apr 15;8:205. doi: 10.1038/s41420-022-00932-7 (PMC9012823; doi:10.1038/s41420-022-00932-7)
Supplement: Supplementary file 5 — Table S2 [file 41420_2022_932_MOESM5_ESM.docx]

**Table S2** Relationship of CHEK1 expression with clinical-pathological characteristics in CCA patients.

| Index | Patients (n = 145) | CHEK1 expression | *P* |
| --- | --- | --- | --- |
| Gender |  |  |  |
| male | 98 | 1.840 ± 0.260 |  |
| female | 47 | 1.829 ± 0.259 | 0.9169 |
| Age (years) |  |  |  |
| ≤ 60 | 89 | 1.845 ± 0.271 |  |
| > 60 | 56 | 1.822 ± 0.241 | 0.3007 |
| Tumor size (cm) |  |  |  |
| ≤ 5 | 59 | 1.774 ± 0.272 |  |
| > 5 | 86 | 1.878 ± 0.242 | 0.0165 |
| Lymph node metastasis |  |  |  |
| with | 45 | 1.905 ± 0.282 |  |
| without | 100 | 1.805 ± 0.243 | 0.0304 |
| TNM stage |  |  |  |
| T1 | 109 | 1.799 ± 0.261 |  |
| T2 | 30 | 1.942 ± 0.231 |  |
| T4 | 6 | 1.973 ± 0.173 | 0.0109 |
