# Supplementary material for: Suppressive effects of umbilical cord mesenchymal stem cell-derived exosomal miR-15a-5p on the progression of cholangiocarcinoma by inhibiting CHEK1 expression
Source: Cell Death Discov. 2022 Apr 15;8:205. doi: 10.1038/s41420-022-00932-7 (PMC9012823; doi:10.1038/s41420-022-00932-7)
Supplement: Supplementary file 6 — Table S3 [file 41420_2022_932_MOESM6_ESM.docx]

**Table S3** Primer sequences of quantitative PCR.

| Target | Sequence |
| --- | --- |
| miR-15a-5p | Forward: 5'-CAGGCCATATTGTGCTGCCTCA-3' |
|  | Reverse: Universal reverse primer |
| U6 | Forward: 5'-GTGCTCGCTTCGGCA-3' |
|  | Reverse: Universal reverse primer |
| cel-miR-39 | Forward: 5'-TCACCGGGTGTAAATCAGCTTG-3' |
|  | Reverse: Universal reverse primer |
| CHEK1 | Forward: 5'-TGCGTTGTAAGATTTATTTTGGCT-3' |
|  | Reverse: 5'-CTTCAGCCCGGTCTTTTTGC-3' |
| GAPDH | Forward: 5'-GACAGTCAGCCGCATCTTCT-3' |
|  | Reverse: 5'-GCGCCCAATACGACCAAATC-3' |
