# Supplementary material for: Suppressive effects of umbilical cord mesenchymal stem cell-derived exosomal miR-15a-5p on the progression of cholangiocarcinoma by inhibiting CHEK1 expression
Source: Cell Death Discov. 2022 Apr 15;8:205. doi: 10.1038/s41420-022-00932-7 (PMC9012823; doi:10.1038/s41420-022-00932-7)

Figure 2D HuCCT1

CHEK1
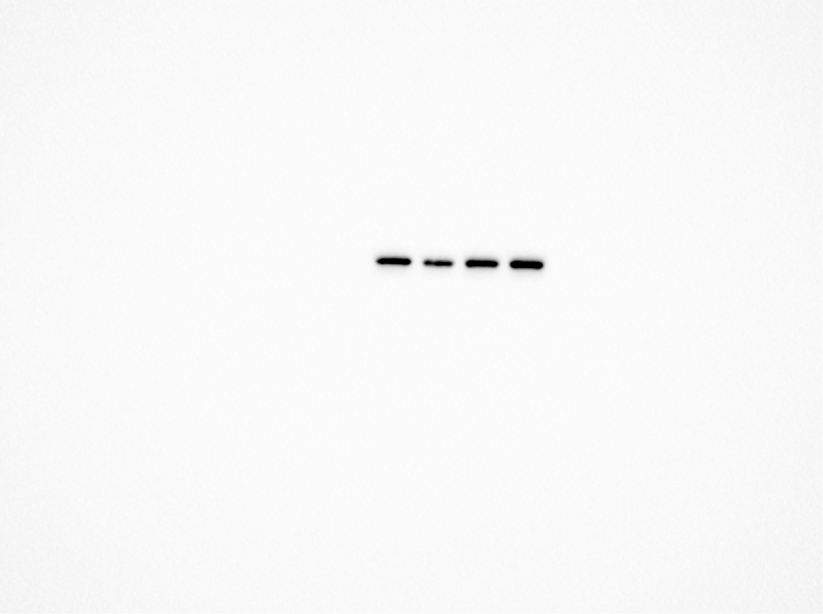


GAPDH
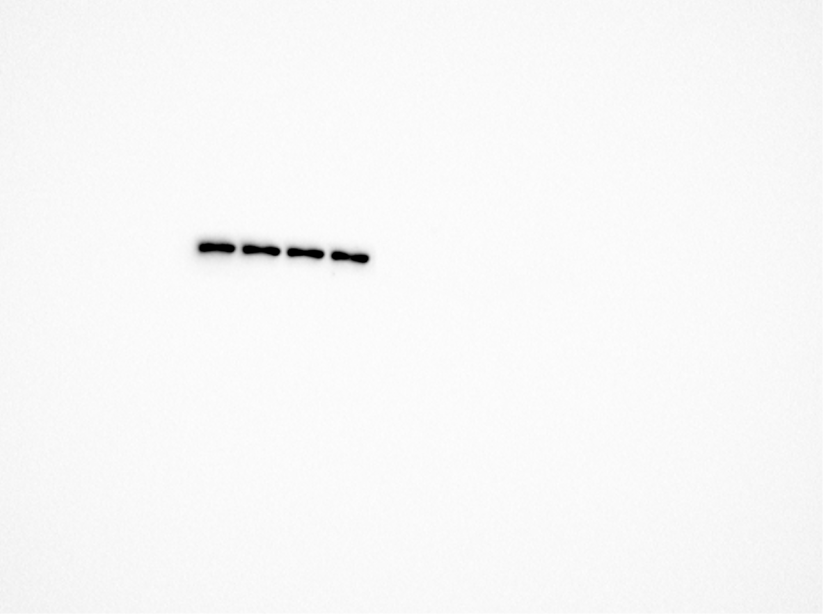


Figure 2D HuH28

CHEK1
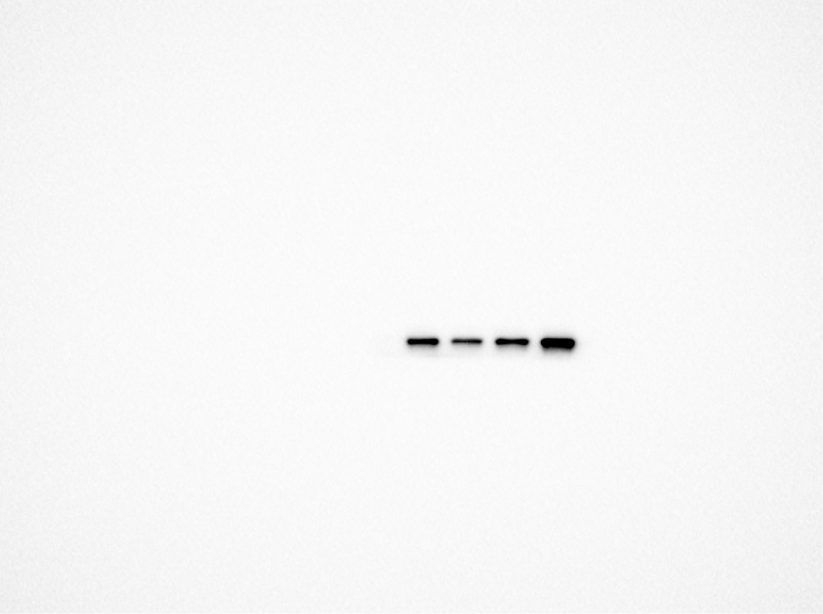


GAPDH
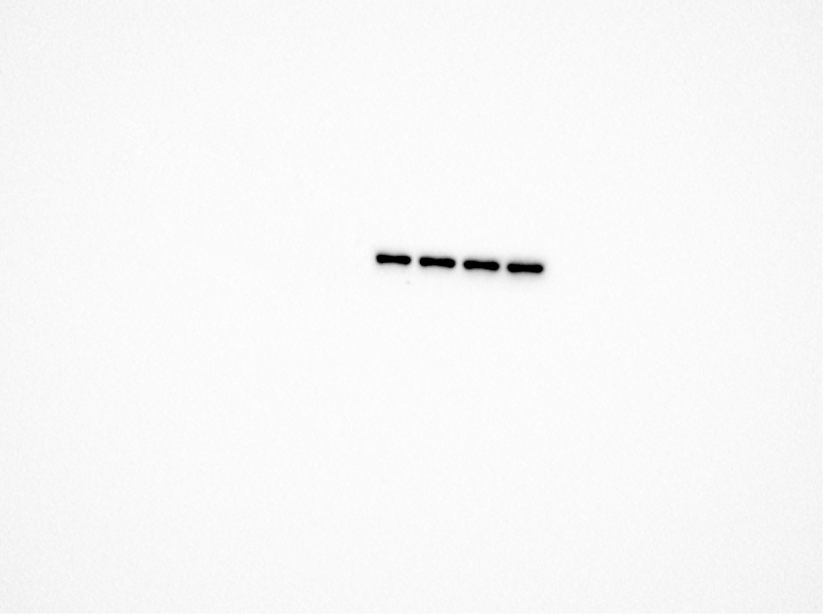


Figure 3A HuCCT1

CHEK1
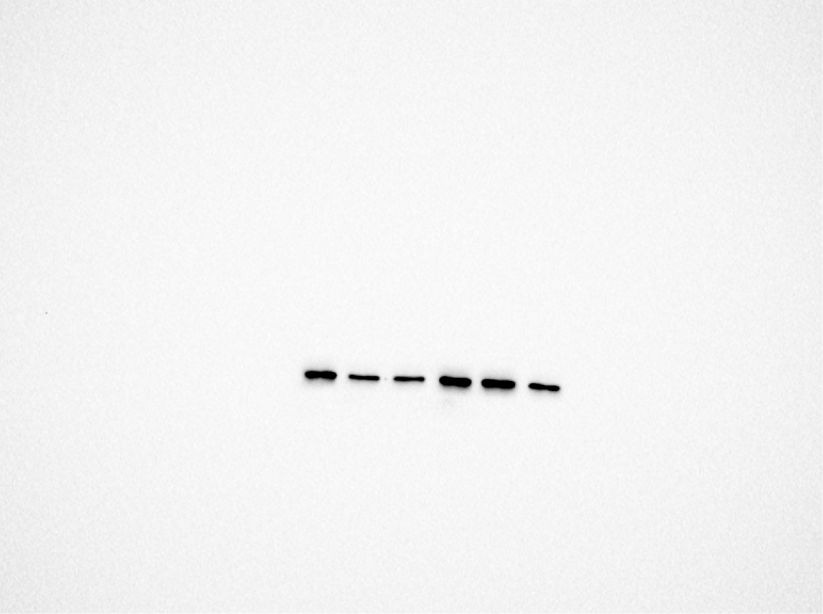


GAPDH
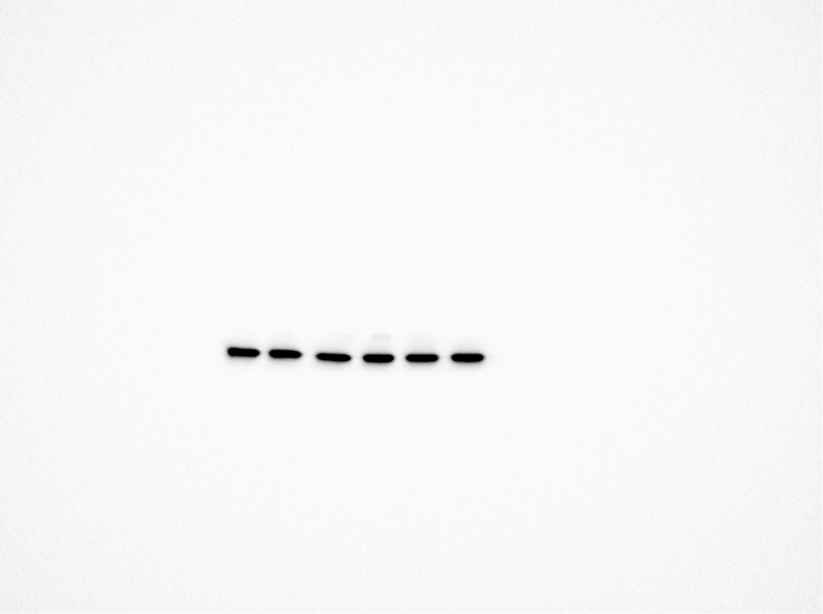


Figure 3A HuH28

CHEK1
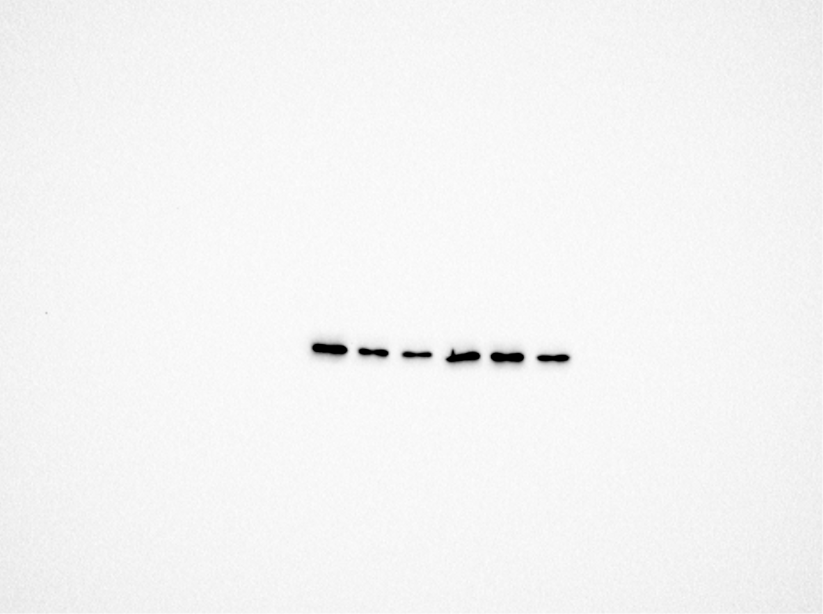


GAPDH
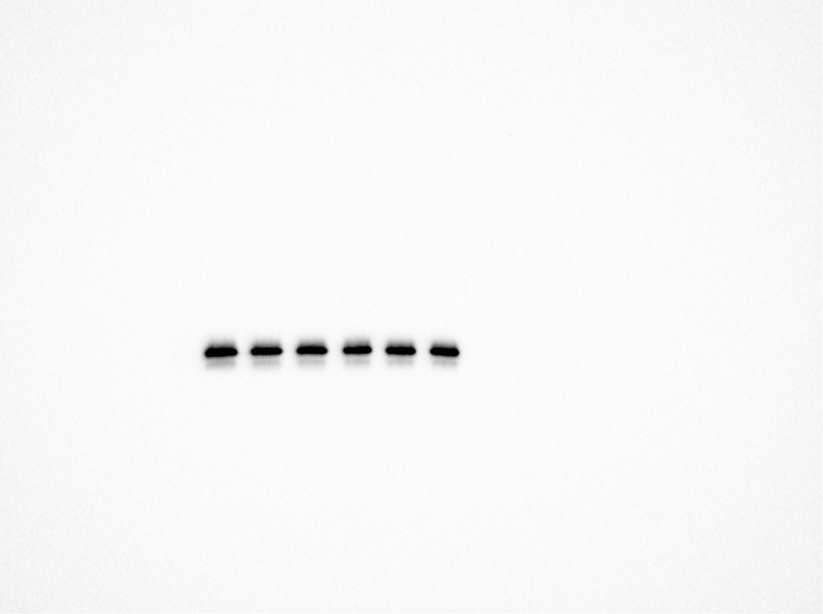


Figure 6E

calnexin
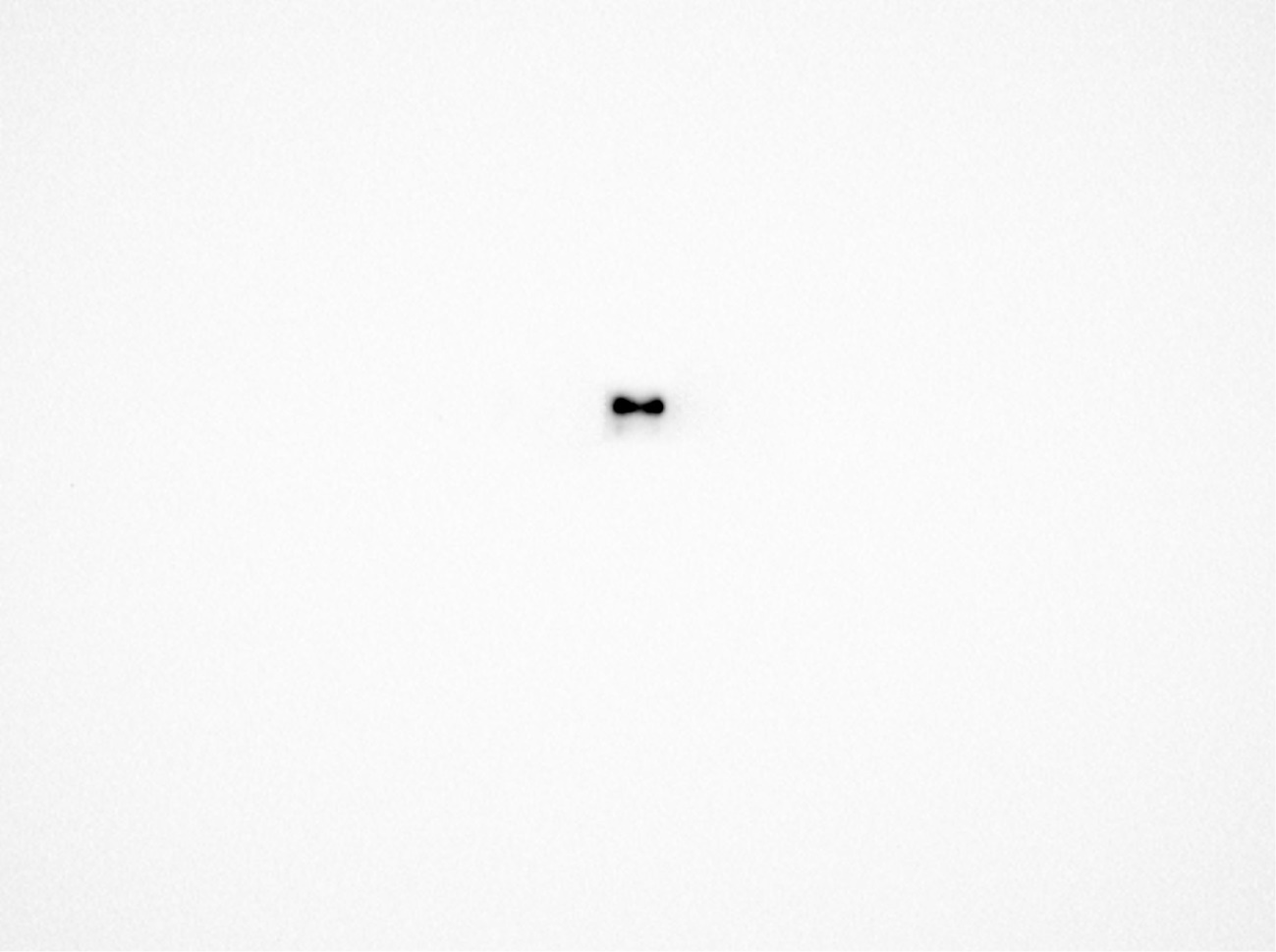


CD63
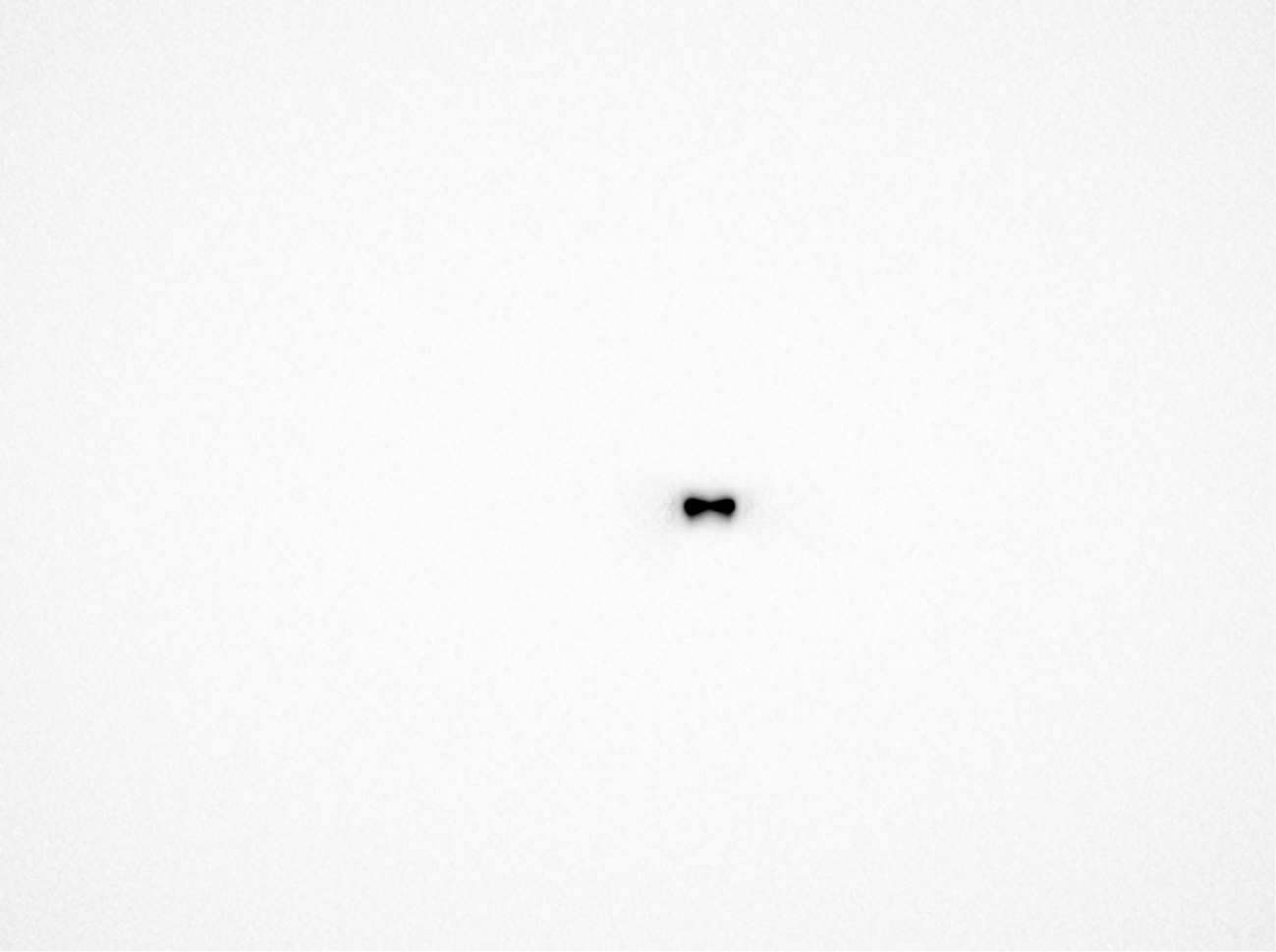


TSG101
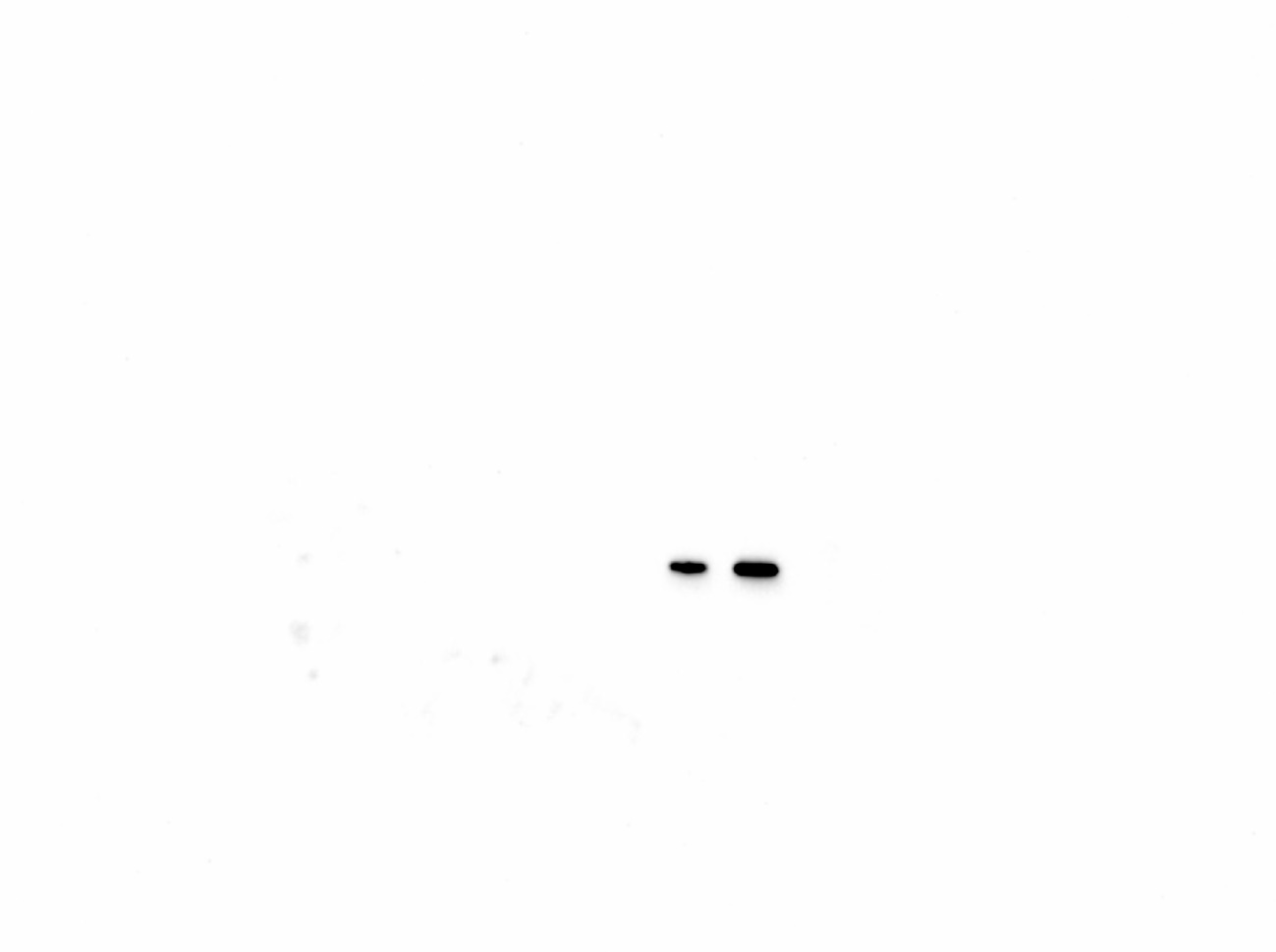


Alix
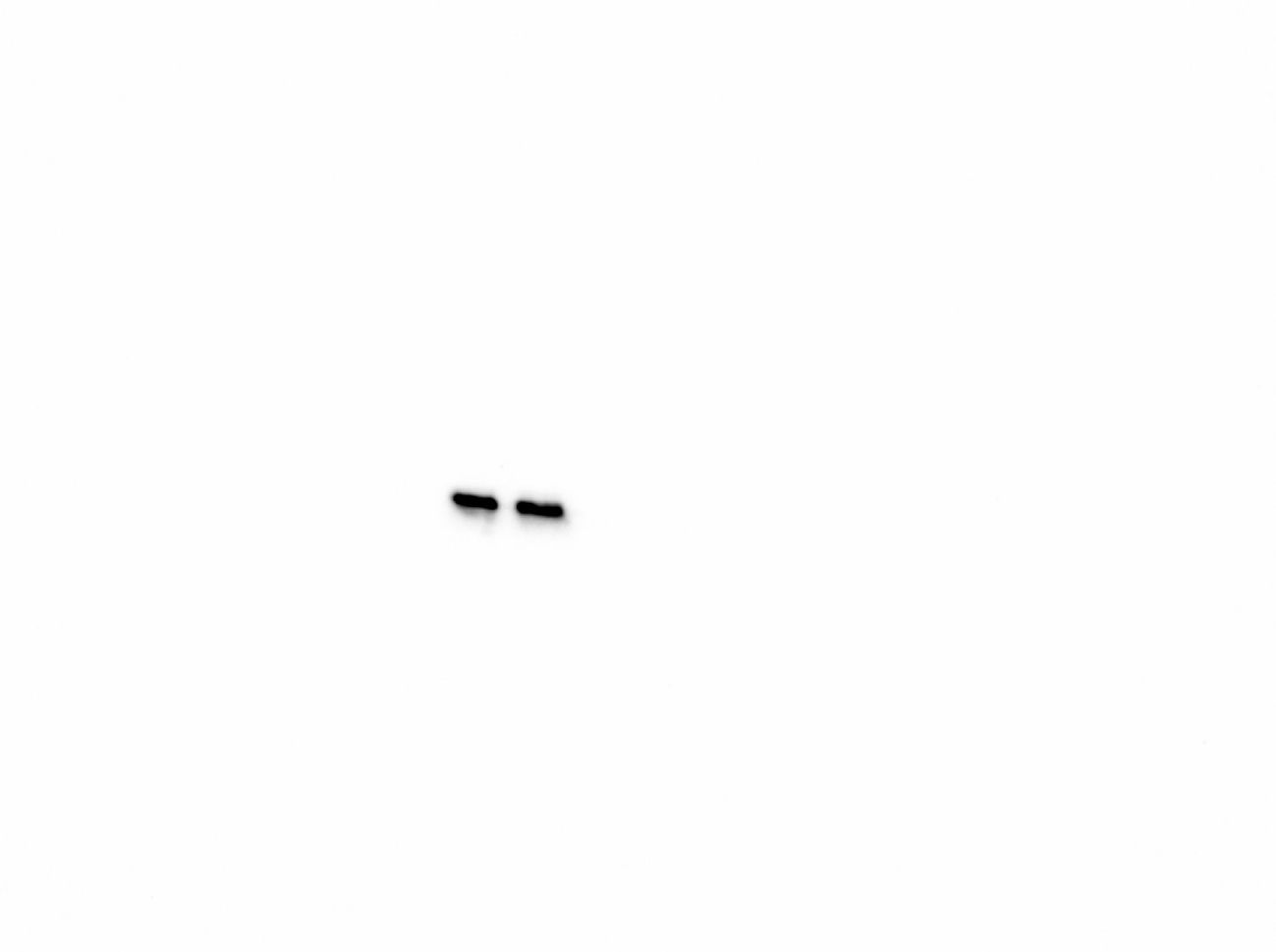

Supplement: Supplementary file 7 — Original Data File [file 41420_2022_932_MOESM7_ESM.docx]
